# Speciation of Chromium in Soil and Sludge in the Surrounding Tannery Region, Ranipet, Tamil Nadu

**DOI:** 10.5402/2011/697980

**Published:** 2011-09-28

**Authors:** Badal Kumar Mandal, Raviraj Vankayala, L. Uday Kumar

**Affiliations:** Trace Elements Speciation Research Laboratory, Environments and Analytical Chemistry Division, School of Advanced Sciences, VIT University, Vellore 632014, India

## Abstract

The distribution and mobility of chromium in the soils and sludge surrounding a tannery waste dumping area was investigated to evaluate its vertical and lateral movement of operational speciation which was determined in six steps to fractionate the material in the soil and sludge into (i) water soluble, (ii) exchangeable, (iii) carbonate bound, (iv) reducible, (v) oxidizable, and (vi) residual phases. The present study shows that about 63.7% of total chromium is mobilisable, and 36.3% of total chromium is nonbioavailable in soil, whereas about 30.2% of total chromium is mobilisable, and 69.8% of total chromium is non-bioavailable in sludge. In contaminated sites the concentration of chromium was found to be higher in the reducible phase in soils (31.3%) and oxidisable phases in sludge (56.3%) which act as the scavenger of chromium in polluted soils. These results also indicate that iron and manganese rich soil can hold chromium that will be bioavailable to plants and biota. Thus, results of this study can indicate the status of bioavailable of chromium in this area, using sequential extraction technique. So a suitable and proper management of handling tannery sludge in the said area will be urgently needed to the surrounding environment as well as ecosystems.

## 1. Introduction

Chromium (Cr) is the seventh most abundant available element on the Earth and 21st in the Earth's crust, with an average concentration of 100 mg/kg. The maximum concentration of chromium allowed in drinking water is 0.10 mg/L due to the toxic effects of Cr(VI) and the potential for oxidation of Cr(III) to Cr(VI) [1]. The discharge of Cr(VI) to surface water is regulated to below 0.05 mg/L by the US EPA [2]. The presence of an excess amount of chromium beyond the tolerable limits makes the land unsuitable for crop growth [3], and the fertility is also adversely affected by wastewater emanating from tanneries [4]. Although the majority of the researchers consider that Cr(VI) is removed by anionic adsorption onto the biomaterials [5], basically the removal mechanism of Cr(VI) by natural biomaterials is adsorption-coupled reduction [6].

As per 2001 census population of the surrounding towns of Ranipet adjacent to Palar river basin that is, Vaniyambadi, Ambur, Pernampattu, and Ranipettai are 103,841; 99,855; 41,323; 36,675, respectively, with 138 tanneries in Vaniyambadi, 83 tanneries in Ambur, 18 tanneries in Pernampattu, and 39 tanneries in Ranipettai. Due to mushrooming of tanneries day by day the study became imperative to check the flow of chromium and other heavy metals in the region. Although some of the tanning industries have been closed down which neither had their own effluent treatment plants nor were connected to a common effluent treatment facility, this hardly made any difference on the ground as tanneries had been discharging effluents for several decades. A large number of small-scale tanneries located within the country do not have access to a common treatment plant and discharge their wastes in open fields or dump them in landfill sites. The use of sludge as a cheap manure in agricultural practices is also not uncommon in this region. These indiscrete methods of disposal contaminate the soil and water providing an easy pathway of Cr in the food chain. 

Plant uptake of chromium accounted for less than 1% of the chromium removed from the soil [7]. Once assimilated by plants, Cr(VI) is readily reduced to Cr(III) [8]. Overall, the addition of organic matter had the strongest influence on chromium mobility [7]. Although bioremediation of Cr(VI) to Cr(III) is a viable cleanup approach, it produces soluble organo-Cr(III) complexes in the environment which depends on the organic ligands [9]. Further, microbial degradation is inactive towards these soluble organo-Cr(III) complexes (citrate-Cr(III) > malate-Cr(III) > histidine-Cr(III) i.e., TCA cycle metabolites). In addition, in mammalian cells the formation of stable DNA-Cr(III) adducts impairs and blocks DNA replication causing mutation [10] which proofs its strong affinity towards organic ligands [11]. Chromate mobility is enhanced in neutral pH groundwater, especially in the presence of competing oxyanions [12–14]. Furthermore, the movement of chromium is associated with its oxide state; that Cr(VI) transforms into more stable Cr(III) is a key progress; organic matter and fertilizers can change the state of heavy metals to reduce its toxicity and promote the removal of chromium [15].

Basic chromium sulfate (BCS) as used in leather tanning processes is not wholly taken up by the hides and skins. Its uptake is limited to 55–70%, and the remaining portion is discharged as waste. Therefore, about 40% of the Cr amount remains in the solid and liquid wastes (especially spent tanning solutions) [16, 17]. The presence of Cr(III) and its salts in the sludge of both the wastewater biological treatment plants or the chemical plants for recycling spent tanning liquors represents an inconvenience for the safe reuse of these sludge and a cost forming factor for their disposal as well as a real threat to environment. Also, tanneries are doing illegal dumping of wastewater and sludge that causes serious environmental pollution.

Tanning is one of the oldest and fastest growing industries in India. There are about 2,000 tanneries located at different centers with a total processing capacity of 600,000 tons of hides and skins per year [18]. Two major sources of Cr contamination are sludge-treated/amended soil [19] and uncontrolled disposal of wastes [20]. It is estimated that in India alone, about 2000–3000 tones of Cr escape into environment annually from the tanning industries, with Cr concentration ranging between 2000 and 5000 mg/L in the aqueous effluent, compared to the recommended permissible limit of 2 mg/L while 0.05 mg/L in drinking water [21]. Hence, in 1995, the Supreme Court of India ordered the closure of hundreds of tanneries in Tamil Nadu for failing to treat their effluents [22]. The Tamil Nadu Pollution Control Board (TNPCB) estimates that about 150,000 tons of solid wastes accumulated over two decades of plant operation were stacked in an open yard (three to five meters high and on 2 hectares of land) on the facility premises.

It is common conception nowadays that the total concentrations of metals in soils are not a good indicator of phytoavailability, or a good tool for potential risk assessment, due to the different and complex distribution patterns of metals among various chemical species or solid phases [17, 23, 24]. Wang et al. [25] reported that correlation was better between plant growth and available Cr than between plant growth and total Cr. It has long been recognized that the soluble, exchangeable, and loosely adsorbed metals are quite labile and hence more bioavailable for plants [26, 27]. Also, clayey soil might have high sorption capacity for Cr than other types of soils [28].

Total metal content of soils is useful for many geochemical applications but the speciation (bioavailability) of these metals is more valuable agriculturally because this form of metal will be bioavailable for plant uptake or extractable to groundwater or animal/human consumption [29–33]. Since mineralogy and chemistry of the soil sample determine the phases or forms of metal salts present, the solubility of these metal salts mostly depends on ionic strength or chemical composition of the extractants. Metal can exist in water soluble, carbonate and sulfide bound, reducible (Fe and Mn oxide bound), oxidisable (organic matter bound), and residual (lattice bound) forms in soils and sludge. Data on metal concentration in each phase are urgently important to access its bioavailability and mobility in underground soil strata under different geochemical environments. 

No single extractant can leach all forms of metal phases in soil, and hence other chemical speciation techniques [31] are not suitable except Sequential Extraction Procedures (SEP). There are several SEP available in the literature [32–37]. Despite the nonselectivity of the reagents used, handling of sediment prior to extraction, sediment-reagent ratio, and length of extraction lead to dubious data collected from SEP [29] and even ends up with inconsistent results using repeatedly the same SEP, these techniques are unanimously accepted and adopted for speciation of metal bound to different salts in soils and sludge. 

There is not enough scientific information about the leaching of Cr from the dump of tannery sludge as well as bioavailable Cr to plants and animals in Ranipet region of Vellore district. Therefore, the present study brought an insight into the much needed information on the fate of Cr on and around the dumping site in the study area using SEP.

The main objectives of the present study were (1) to study the viability of dumping tannery sludge in an open place for a long time; (2) to study the percentage of bioavailable chromium present in the sludge and the effluent; (3) to study the percentage of leachable chromium present in the sludge and effluent contaminating ground water; (4) to study the percentage of immobilisable chromium present in the sludge and effluent for long run.

## 2. Materials and Methods

### 2.1. Reagents

All chemicals were of analytical grades. Potassium dichromate, diphenylcarbazide, cupferron, sulphuric acid, chloroform, nitric acid, and 30% hydrogen peroxide were purchased from SD Fine Chemicals (Bombay, India) and Qualigens (GlaxoSmithKline, Bombay, India). All samples were prepared using double distilled water.

### 2.2. Protocol

Samples (sludge, effluent and soils) were collected from in and around Ranipet Common Effluent Treatment Plant area near Walajapet (about 100 miles from Chennai), paddy field and agricultural fields. Sequentially extracted, acid and microwave-digested samples were analyzed by UV-Visible Spectrophotometer (Schimadzu 8500 II, Tokyo, Japan). Standard Colorimetric Method 3500-D of American Public Health Association (APHA) for Cr analysis was adopted in the present study [38]. For acid digestion EPA-3050B method was performed, and for microwave-assisted digestion of samples EPA 3052 method was adopted in the present study for soil and sludge samples.

### 2.3. Sample Collection

The site chosen for the present study was the Ranipet Common Effluent Treatment Plant (RCETP) area near Walajapet. Tannery is the basic unit of leather industry, which requires large amount of water and chemicals and major parts of it are discharged as effluent. There are more than 200 Tanneries and finished leather goods manufacture industries located in the above specified area. Different types of samples such as sludge samples (S1–S14, *n* = 13) and effluent samples (*n* = 7) from different tanneries located near to the Ranipet Common Treatment Plant and soil samples (*n* = 30) were collected from each location at three different depths. The samples at each point were collected at 10–20, 30–40, and 45–60 cm, respectively. Sludge samples (S1–S9) were collected surrounding REETP, and sludge samples (S10–S13) were collected from the four corners of the rectangular shaped core sludge dumping spot. Basically S10 and S11 samples were collected from recently dumping spot, and S12 and S13 samples were collected from other side of the recently dumping area. All a-marked soil samples were collected at a depth of 10–20 cm from the surface, b-marked samples collected at a depth of 30–40 cm, and c-marked samples collected at a depth of 45–60 cm, respectively.

### 2.4. Sample Preparation

It was found that in optimum conditions of sequential extraction of soil the air-dried soil or drying of soil at 20–30°C should be used for effective determination of Cr in soil eluates. In higher drying temperatures Cr contained in soil created probably sparingly soluble compounds, which did not get easily into eluates with hydroxylamine hydrochloride (NH_2_OH·HCl) and hydrogen peroxide (H_2_O_2_), causing distortion pattern of the actual Cr distribution in various metals fraction [39]. That's why, after the collection of soil samples they were air-dried at 30°C in a hot air oven until a constant weight was achieved. Also, Kalembkiewicz and Sočo [39] found that with an increase of grain diameter (>0.25 mm) the Cr concentration in eluates decreased. Thus, grain diameter of soil used for leaching should not be larger than 0.25 mm. In the present study grain diameter of all samples were below 0.25 mm. Then they were digested with nitric acid and hydrogen peroxide at 100°C for 2 hrs. Then digested sample was filtered using Supor membrane filters (0.2 mm pore size, Sigma-Aldrich, MO, USA), and the filtrate was taken for the spectroscopic analysis. Sludge samples were subjected to SEP [40, 41]. Effluent samples were centrifuged and filtered before the digestion and analysis.

### 2.5. Analytical Procedure

Chemical speciation determines the potential environmental mobility and bioavailability of heavy metals in soil. Information about their physicochemical forms is thoroughly required for understanding the environmental behaviour (mobility, bioavailability, pathways) of heavy metals in soil. Sequential extraction procedures or fractionation have been widely applied to soils to know their chemical phase association [42]. Change in oxidation state, soil pH, temperature, redox potential, soil organic matter decomposition, and so forth have a profound effect on its essentiality, toxicity, and bioavailability.

The sequential extraction procedure consists of a series of chemical extractants, each being more drastic in action and of different nature than the previous one [43]. As no reagent is single phase specific in element removal, sequentially extracted phases are “operationally” defined. The various selected phases include water soluble, exchangeable, carbonate and sulfide bound, reducible (Fe and Mn oxide bound), oxidisable (organic matter bound), and residual (lattice bound). All phases except the residual one become available to biota under changing environmental conditions. To estimate the amount of Cr bound to different phases in soils and sludge, SEP was carried out using the following steps.

#### 2.5.1. Collection of Water-Soluble Fraction

2.0 grams of dry sample was weighed and taken in a 50 mL capped centrifugable bottle. The soil was dissolved in 50 mL water and mechanically shaken for 30 min. Then it was centrifuged for 15 min at 3335 × g (REMI C-24BL, REMI Instruments Ltd, Mumbai, India) and the water-soluble fraction was collected. The residue was used for the collection of exchangeable fraction.

#### 2.5.2. Collection of Exchangeable Fraction

50 mL of 0.5 M Mg(NO_3_)_2_ was added to the above residue and mechanically shaken for 30 min. Then it was centrifuged for 15 min at 3335 × g, and the exchangeable fraction was collected. The residue was used for the collection of carbonate and sulfide bound fraction.

#### 2.5.3. Collection of Carbonate and Sulfide Bound (Weakly Absorbed/Acid Leachable) Fraction

50 mL of 1 M NaOAc was added to the residue and mechanically shaken for 5 hrs. Then it was centrifuged for 15 min at 3335 × g, and the carbonate and sulfide bound fraction was collected.

#### 2.5.4. Collection of Fe and Mn Oxide Bound (Reducible) Fraction

50 mL of 0.08 M NH_2_OH·HCl was added to the residue left after collection of carbonate and sulfide bound fraction and mechanically shaken for 6 hrs. Then it was centrifuged for 15 min at 3335 × g, and the Fe and Mn oxide bound fraction was collected.

#### 2.5.5. Collection of Organic Matter Bound (Oxidisable) Fraction

10 mL of H_2_O_2_ was added to the residue left after collection of reducible fraction and heated for 2 hrs at 85°C. Then it was centrifuged for 15 min at 3335 × g, and the organic matter bound fraction was collected.

#### 2.5.6. Collection of Lattice/Mineral Matrix Bound (Residual) Fraction

5 mL of concentrated HNO_3_ was added to the residue left after collection of oxidisable fraction and heated for 2 hrs at 105°C. Then it was centrifuged for 15 min at 3335 × g, and the mineral matrix phase was collected.

All samples were filtered through Supor membrane filters (0.2 mm pore size, Sigma-Aldrich, MO, USA) after sequential extraction and digestion. The samples were analyzed by UV-Visible Spectrophotometer (Schimadzu 8500 II, Tokyo, Japan). Standard Colorimetric Method 3500-D of American Public Health Association (APHA) for Cr analysis was adopted in the present study [38]. Standard Colorimetric Method 3500-D (APHA) consists of five steps depending on the nature of the samples. The present study used step 1 for calibration curve, step 3 for removal of interferences with cupferron, and step 5 for colour development and measurement of Cr content at 540 nm in all samples.

EPA 3050B method was used for the digestion of soil and sludge samples. In brief, for the digestion of samples, a representative 1-2 gram (wet weight) or 1 gram (dry weight) sample was digested with repeated additions of nitric acid (HNO_3_) and hydrogen peroxide (H_2_O_2_) and finally was diluted to 50 mL using doubled distilled water.

For microwave-assisted digestion of samples, a microwave digestion system (EM-S1563) from Sanyo Corporation, Mumbai, India with a rotor for fourteen Teflon digestion vessels HP-500, was used for sample digestion. Approximately 1.0 g of dry, finely powdered soil/sludge sample was weighed into a dry, clean Teflon digestion vessel. Two mL of MilliQ water, 3 mL of concentrated HNO_3_, and 2 mL of H_2_O_2_ were added. The vessel was closed, placed into the rotor, and tightened. The loaded rotor having maximum seven vessels per single time was placed into the microwave oven. The microwave conditions for three stage digestions were as follows. For stages 1–3 the values of power (%) (600 W) were 100 s, PSI were 70, 120, 150; RAMP (min) were 20, 10, 10, and Hold (min) were 10 s, respectively. After cooling for 30 min, the vessels were opened carefully. Each digested solution was transferred quantitatively to a 50 mL volumetric flask and made up to the mark with Milli-Q water. Finally it was filtered through a millipore membrane (0.45 *μ*m) and kept in plastic container for analysis.

### 2.6. Statistical Analysis

All statistical works were done using StatView statistical software (StatView, SAS Institute Inc., Second Edition, USA, 1998). Student's* t*-test was used to test the null hypothesis that the means of the two groups are the same or not, and a significant *P* value (*P* < 0.05) means are not the same. Statistical significant values were considered in all cases as *P* < 0.05.

## 3. Results and Discussion

### 3.1. Total Chromium Concentration

The critical soil concentration or the critical load is defined as the range of values above which toxicity is considered to be possible. Kabata-Pendias and Pendias [44] considered 75–100 mg/kg as critical value/load for chromium in soils. The concentration in the lowest soil horizon is commonly used to represent the natural background value, which is representative of individual soil profile [45].

For total Cr content all soils and sludge samples were digested by both EPA 3050 method and microwave digestion method. Sludge samples collected from the dumping site contained 377–1052 *μ*g Cr/g sludge (*n* = 4) after acid digestion, whereas 413–1213 *μ*g Cr/g sludge (*n* = 4) after microwave digestion, whereas sludge samples collected from the tannery contained 492–2941 *μ*g Cr/g sludge (*n* = 9) after acid digestion, whereas 490–3540 *μ*g Cr/g sludge (*n* = 9) after microwave digestion (raw data not shown). With respect to total Cr obtained in sludge samples by SEP the recovery of Cr was within the range of 96.2–114% by acid digestion and 87.2–108% by microwave digestion (raw data not shown). Soil samples collected from nearby agricultural lands contained 184–562 *μ*g Cr/g soil (*n* = 30) after acid digestion whereas 188–568 *μ*g Cr/g soil (*n* = 30) after microwave digestion, which is much lower than that of sludge samples. Similarly, with respect to total Cr obtained in soil samples by SEP, the recovery of Cr was within the range of 91.4–100% by acid digestion and 90–98.5% by microwave digestion (raw data not shown), respectively. Both methods gave almost similar recovery of Cr in sludge and soil samples in the present study.

The recovery results were comparable to the results of Chaudhary and Banerjee [42] (87.89 to 114.14%). In industrially contaminated land, 63–147% recovery was reported for Cr [46] which had a broad range of recovery compared to our results, whereas the metal concentration in the sludge was higher compared to that reported in Oake et al. [47].

Effluent samples collected from the tanneries contained about 7.5–45 *μ*g Cr/L effluent (*n* = 7) (raw data not shown). This result suggests that a major fraction of Cr in soil might have leached and percolated to deeper layer which causes contamination of groundwater and subsequently highly prone to mobilization to the surrounding water layers as well as available to biota. The concentration of total chromium in the soil samples has decreased with the increase in the depth with few exceptional cases. Similarly, the concentration has decreased with the increase in distance between the dumping site and the sampling spot surrounding the agricultural field. 

To get insights on Cr distribution in soils and sludge collected from the study area, the results of SEP study of sludge and soil samples are analyzed as fraction wise.

### 3.2. Water-Soluble Fraction

The concentration of Cr in water-soluble fraction was 19.8% in soils and 7.11% in sludge samples of the total Cr content (Tables 1 and 2). It clearly indicates that soils contain higher percentage of water-soluble Cr than that of Cr in sludge, and hence sludge is less prone to rain water leaching compared to soils. Although water-soluble fraction in sludge is comparatively less than that in soil, it is not recommendable to store tannery sludge in an open place for long run.

### 3.3. Exchangeable Fraction

The concentration of Cr in the exchangeable form decreases from the top to the bottom layer of the soil strata and decreases with decrease in pH. Thus it can be interpreted that, in cases where pH is acidic, the concentration of exchangeable fraction is less than in those having alkaline pH. This may be due to Cr(VI) reduction to Cr(III) at acidic pH, which is the immobile form. Similar findings were reported by Cary et al. [48], and the reduction of Cr(VI) was more rapid in acid than in alkaline soils, and hence soluble Cr(III) was rapidly transformed to its mobile forms due to complex formation with humus substances present in the soils immediately after reduction. Another report [46] found that solubility of Cr(III) decreased as the solution pH increased above 5 and eventually Cr(III) showed complete precipitation at pH 5.5. In this study, exchangeable Cr was 6.8% and 10.9% of total Cr content in soils and sludge, respectively, (Tables 1 and 2), which was comparable to other reports [42, 49–51].

### 3.4. Carbonate and Sulfide-Bound Fraction (Weakly Absorbed/Acid Leachable Fraction)

Trace metals may coprecipitate with carbonate and sulfide both inorganically and biologically. This fraction is highly susceptible to pH changes [23]. The carbonate bound fraction accounted for a relatively small amount of total Cr concentration in soils (9.7%) compared to a relatively higher amount of total Cr concentration in sludge (12.2%) and decreased slightly downwards from the soil surface in the present study (Tables 1 and 2). These results clearly matched with the results of previous workers [42, 51]. Although acid leachable fraction was about 10% of total Cr content, it still possesses threat in acidic soils or acid-contaminated environment.

### 3.5. Reducible Fraction (Iron and Manganese Oxide Bound Fraction)

Chromium(III) oxidation in soil increased with Mn(IV) oxides content of the soil [52]. Under certain circumstances Cr(III) could be oxidized to more mobile and toxic Cr(VI) in soils containing Mn oxide [53, 54]. Non-humic organic substances such as carbohydrates and proteins [55], and Organic matter can reduce Cr(VI) in soils [56, 57]. Hence iron and Mn oxides can retain substantial amounts of metals and play an important role in controlling the fate of heavy metal mobility in the environment [58]. Theoretically, this fraction represents the content of metal bound to iron and manganese oxides that would be released if the sediment experienced more reducing environment [59]. In the present study, the concentration of Cr in this phase was 31.3% in soils and 6.3% of the total Cr content in sludge (Tables 1 and 2). On an average the amount of chromium in Fe- and Mn-oxide bound fraction was 69.1 *μ*g/g of sludge (Table 1). This fraction of Cr is not bioavailable to plants and biota, that is, immobile in nature. 

Higher concentration of Cr in the reducible phase suggests close association of it with Fe-Mn oxides and *in situ* changes in pH or redox potential could easily mobilize Cr associated with reducible phase. The high percentage of total Cr in this fraction proposes that Fe-Mn bound phase acts as a scavenger of Cr in polluted soils. So the mobility of this fraction should be monitored critically. Otherwise this portion will spread over throughout underground basin and may contaminate easily surrounding areas under variable environmental settings.

### 3.6. Oxidizable Fraction (Organic-Bound Fraction)

Normally tannery effluents, sludge, and soils surrounding these tanneries are rich in organic matters due to lack of treatment technologies of wastes generated in tanneries. Trace metals bound to various forms of organic matter (living organisms, detritus coating on mineral particles) were extracted in this fraction. In the present study, organic matter bound fraction was 616 *μ*g/g of sludge, and oxidisable Cr in sludge was 56.3% of the total Cr content which was higher than the other profiles, but soils had 14.1% oxidisable Cr of the total Cr content (Tables 1 and 2). These variations seem to be controlled by total organic carbon content and can be ascribed to the fact that organic matter present in the soil acts as an electron donor and thus provides favourable conditions for the reduction of Cr(VI) to Cr(III) [46]. The organic matter consumed by microbial activity can cause release of organic acids due to degradation process and thus a decrease in pH. This would affect speciation of Cr, as a lower pH would favour Cr(III). The Cr(III) so formed has a capability to form soluble organic complexes, which are mobile in nature and show their presence as high organic bound Cr in this fraction. The decrease in carbon content with depth causes decrease of complexation of Cr and hence its concentration decreases in this fraction. This concept clearly clarifies the presence of high organic bound fraction in contaminated soils near mining area (13%) [33] and in industrially contaminated land (19%) [50], since all these areas contain less organic carbon that is rich in inorganic materials. As high as 70% of total Cr in oxidisable phase has been found in tannery wastewater irrigated land [60]. The high percentage of Cr in sludge indicates that sludge is richer in organic matter than that of soils which is highly reasonable due to absorption of fatty acids, amino acids as well as contamination of muscles, and skin residues during the settling of effluents.

### 3.7. Residual Fraction (Lattice-Bound Fraction)

The Cr concentration in mineral matrix was 123 *μ*g/g of sludge, which was fully immobile in nature for long run. The lower percentages of the total Cr content in residual fraction in soils (13%) and sludge (11.3%) were in strong agreement with the results obtained by Rauret et al. [61] (Tables 1 and 2). This phase is a chemically inert fraction and may not be easily accessible to biota. So, this fraction of Cr does not pose any threat to environment. Although the easily available exchangeable form in this sludge constitutes only 10.9%, the actual Cr content corresponding to this form may be elevated for the disposal of the sludge in the environment. In contaminated sites the concentration of Cr was found to be higher in the reducible phase in soils (31.3%) and oxidisable phases in sludge (56.3%) (Tables 1 and 2).

Typically metals of anthropogenic inputs tend to reside in the first four fractions, and metals found in the residual fraction are of natural occurrence in the parent rock [30]. So speciation of chromium is essential for research on Cr mobility. A few reports on SEPs informed combining the exchangeable and carbonate-bound fractions in a single step [34] or dividing the Fe and Mn oxide fractions into the amorphous oxyhydroxides and crystalline oxides [43] or including EDTA extractable, moderately reducible, and strongly reducible fractions [62] or even nine fractions for testing waste amended and agriculturally polluted sediments [63]. Although fractions and chemicals used were different for different studies using SEPs all [29, 30, 32, 64] found the same order of extraction of metals as Cd > Pb > Zn *≈* Cu > Mn > Ni > Fe *≈* Cr. Other important findings were that metals collected for fractions 1–3 were less than expected and for fractions 4-5 were greater than expected. In the present study, the sum of first three fractions is 36.3% in soils and 30.2% in sludge, whereas the sum of 4th and 5th fractions is 45.4% in soils and 62.6% in sludge. Although these results supported the above statement, more thorough research will be needed to address whether metals such as Cu, Mn, Ni, Pb, V, and Zn that released in the first three fractions were reattaching to the newly available sites of the next fraction [37] with varying soil composition and the nature of the metal. Consequently the fractional quantification will be skewed toward lower than real results for the fractions related to first three fractions and skewed toward higher than real results for 4th and 5th fractions [30, 37].

**Figure 1:**
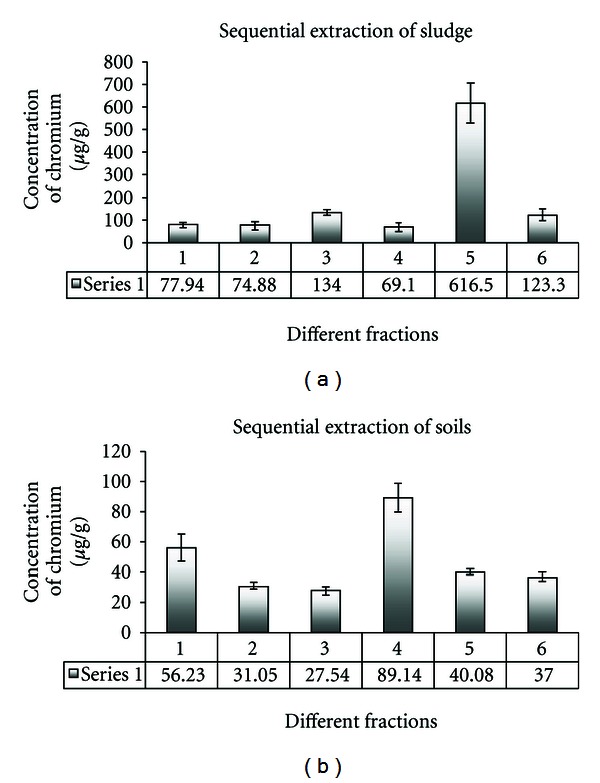
Bar diagram of average concentrations of Cr (in *μ*g/g) in different sequential extracted fractions (along *Y*-axis) versus types of extractants (along *X*-axis) in (a) tannery sludge and (b) agricultural soils. (1) Water-soluble fraction, (2) exchangeable fraction, (3) carbonate and sulfide bound fraction, (4) Fe and Mn oxide bound fraction, (5) organic matter bound fraction, and (6) mineral matrix bound fraction.

The present study gave some important findings. An increasing trend was observed when average concentrations of Cr were plotted against different sequential fractions in sludge samples. It clearly shows that the value of organic matter and sulfide bound Cr (fraction 5) was maximum in the sludge samples (Figure 1(a)), but it was not conclusive when average concentrations of Cr were plotted against different sequential fractions in soil samples (Figure 1(b)). In the case of soil samples the highest value of Fe-Mn bound Cr was found among other fractions. Higher concentration of Cr in the reducible phase suggests close association of it with Fe-Mn oxides and *in situ* changes in pH or redox potential could easily mobilize Cr associated with reducible phase. The high percentages of total Cr in this fraction (31.3% in soil and 6.3% in sludge) show that Fe-Mn bound phase acts as scavenger of Cr in polluted soils. The present result is highly comparable to the result of Chaudhary and Banerjee [42]. The present result indicates that iron rich soil can hold Cr which will be available to plants and biota under favorable environmental conditions. 

Also, this study shows that about 63.7% of total Cr is mobilisable and 36.3% of total Cr is nonbioavailable in soil, whereas about 30.2% of total Cr is mobilisable and 69.8% of total Cr is nonbioavailable in sludge. But under oxidisable conditions 14.1% of total Cr in soil and 56.3% of total Cr in sludge (fraction 5), that is, organic bound Cr are mobilisable due to degradation of organic substances by microorganisms (Tables 1 and 2). 

In addition, Cr(III) will form soluble complexes in acidic conditions, which are mobile in nature. Only 13% of total Cr was in the residual phase in soil (fraction 6), and 11.3% of total Cr was in the residual phase in sludge (fraction 6), which are stable and absolutely immobile in nature. Inversely, on an average water-soluble fraction, exchangeable fraction and carbonate-bound fraction contained 77.9, 74.9 and 134 *μ*g/g (*n* = 13) in sludge samples, respectively (Table 1). This easily leachable and mobilisable Cr is easily available to plant and biota intake as well as contamination of groundwater.

## 4. Conclusions

The results of the present study clearly indicate that there is a possibility of Cr percolation into the ground water, which may lead to many health hazards and disturb the surrounding livelihoods. Also, the dumping of tannery sludge in an open land is not safe for ecosystem due to degradation with time under normal environmental conditions. The results of Cr content and speciation profile of both the sludge and soils indicate that it may not be safe to dispose the sludge on barren land and in water bodies as well as land filling. Thus one of the viable alternatives is the effective recovery of Cr from the sludge before dumping in an open field. Since there is discrepancy in results due to nonselective reagents and other uncertainties, proper selection of reagents coupled with XRD would be promising research areas for understanding of metal bound chemistry in soils and sludge. So a suitable and proper management of handling tannery sludge in the said area is urgently needed to save the surrounding environment as well as ecosystems.

## Figures and Tables

**Table 1:** Concentration (Ave ± Stdev, *n* = 3 (in *μ*g/g)) of different forms of Cr in tannery sludge samples with sequential extraction.

S. No	Water soluble	Exchangeable	Carbonate & sulfide	Reducible fraction	Oxidisable fraction	Residual fraction	Total Cr
S1	18.20 ± 0.36	194.2 ± 1.11	145.6 ± 1.20	24.27 ± 0.32	2275 ± 3.84	121.3 ± 0.92	2779 ± 4.5
S2	52.79 ± 0.48	49.27 ± 0.43	172.5 ± 1.40	21.11 ± 0.17	586.5 ± 1.61	190.0 ± 0.87	1073 ± 2.1
S3	75.12 ± 0.53	87.84 ± 0.55	179.1 ± 0.87	13.80 ± 0.07	577.9 ± 2.13	115.5 ± 1.02	1049 ± 1.9
S4	70.15 ± 0.44	15.30 ± 0.13	159.4 ± 0.82	181.1 ± 1.43	797.1 ± 2.34	31.8 ± 0.21	1255 ± 1.5
S5	57.36 ± 0.37	82.60 ± 0.47	91.78 ± 0.43	18.35 ± 0.06	186.1 ± 1.82	43.0 ± 0.37	479 ± 1.1
S6	31.09 ± 0.21	163.2 ± 1.33	121.0 ± 0.73	26.43 ± 0.12	77.73 ± 0.75	330.3 ± 3.34	757 ± 1.5
S7	150.6 ± 0.73	13.79 ± 0.23	97.84 ± 0.64	106.6 ± 0.71	47.03 ± 0.64	78.39 ± 0.46	494 ± 0.8
S8	95.37 ± 0.56	47.69 ± 0.51	59.61 ± 0.45	38.15 ± 0.38	2578 ± 4.31	268.2 ± 1.63	3087 ± 5.3
S9	68.58 ± 0.49	40.00 ± 0.32	133.7 ± 0.94	144.0 ± 0.88	77.44 ± 0.74	42.86 ± 0.44	500 ± 1.2
S10	149.5 ± 1.13	58.53 ± 0.61	145.6 ± 0.56	87.85 ± 0.82	627.5 ± 2.24	31.37 ± 0.65	1100 ± 2.5
S11	43.53 ± 0.33	180.3 ± 0.97	236.3 ± 1.62	205.2 ± 1.26	46.64 ± 0.31	155.4 ± 1.12	867 ± 1.6
S12	86.23 ± 0.62	9.58 ± 0.07	119.8 ± 1.53	9.58 ± 0.03	59.88 ± 0.27	131.7 ± 0.91	416 ± 0.9
S13	114.8 ± 0.37	31.20 ± 0.08	81.12 ± 0.72	21.22 ± 0.05	78.00 ± 0.44	62.40 ± 0.38	388 ± 0.5

**Table 2:** Concentration (Ave ± Stdev, *n* = 3 (in *μ*g/g)) of different forms of Cr in soil samples with sequential extraction.

S. No	Water soluble	Exchangeable	Carbonate & sulfide	Reducible fraction	Oxidisable fraction	Residual fraction	Total Cr
1a	51.85 ± 0.34	29.62 ± 0.20	17.28 ± 0.30	98.76 ± 0.92	55.55 ± 0.32	64.81 ± 0.67	317.9 ± 2.1
1b	83.29 ± 0.61	36.74 ± 0.60	14.69 ± 0.40	102.8 ± 1.20	52.05 ± 0.43	42.87 ± 0.41	332.5 ± 2.8
1c	44.48 ± 0.21	23.47 ± 0.05	13.59 ± 0.06	98.86 ± 0.58	49.43 ± 0.17	24.70 ± 0.04	254.6 ± 1.9
2a	80.29 ± 0.42	29.29 ± 0.15	13.38 ± 0.22	105.8 ± 0.73	42.57 ± 0.21	36.49 ± 0.35	307.8 ± 2.7
2b	49.11 ± 0.33	36.83 ± 0.30	19.64 ± 0.33	115.4 ± 0.72	30.69 ± 0.41	39.90 ± 0.06	291.6 ± 0.9
2c	81.04 ± 0.61	12.27 ± 0.60	18.41 ± 0.16	103.1 ± 0.80	36.83 ± 0.33	61.39 ± 0.61	313.1 ± 2.1
3a	57.09 ± 0.23	35.98 ± 0.45	17.36 ± 0.23	107.9 ± 0.90	34.11 ± 0.31	68.23 ± 0.63	327.7 ± 1.7
3b	34.58 ± 0.21	44.33 ± 0.21	16.00 ± 0.31	108.3 ± 0.32	36.94 ± 0.42	36.94 ± 0.32	277.1 ± 1.1
3c	22.55 ± 0.09	45.11 ± 0.38	42.60 ± 0.62	112.7 ± 0.56	43.85 ± 0.26	25.06 ± 0.12	291.9 ± 0.7
4a	38.58 ± 0.08	13.78 ± 0.30	16.50 ± 0.24	26.18 ± 0.32	44.78 ± 0.51	31.00 ± 0.05	170.9 ± 0.8
4b	47.52 ± 0.11	32.51 ± 0.80	27.59 ± 0.14	12.50 ± 0.04	28.13 ± 0.44	56.29 ± 0.22	204.5 ± 1.3
4c	33.46 ± 0.05	11.15 ± 0.10	26.77 ± 0.72	94.82 ± 0.71	25.93 ± 0.07	25.10 ± 0.80	217.3 ± 1.4
5a	44.24 ± 0.53	34.41 ± 0.70	57.76 ± 0.43	110.7 ± 0.48	30.72 ± 0.62	18.43 ± 0.22	296.2 ± 1.5
5b	32.08 ± 0.33	34.55 ± 0.32	25.91 ± 0.41	103.7 ± 0.83	49.35 ± 0.70	2.77 ± 0.01	240.3 ± 2.0
5c	44.05 ± 0.42	31.81 ± 0.27	14.68 ± 0.32	99.11 ± 0.37	30.59 ± 0.17	48.94 ± 0.42	269.2 ± 0.7
6a	37.34 ± 0.06	9.03 ± 0.20	25.20 ± 0.13	17.01 ± 0.03	42.00 ± 0.51	35.00 ± 0.04	218.9 ± 1.4
6b	76.20 ± 0.23	29.49 ± 0.61	40.56 ± 0.73	307.2 ± 1.31	67.60 ± 0.48	30.70 ± 0.18	551.9 ± 2.3
6c	116.7 ± 0.92	26.20 ± 0.39	17.86 ± 0.44	78.60 ± 0.43	29.71 ± 0.32	29.77 ± 0.27	298.9 ± 1.7
7a	36.63 ± 0.07	29.37 ± 0.26	40.30 ± 0.72	102.5 ± 0.72	30.53 ± 0.05	39.69 ± 0.62	279.1 ± 1.5
7b	32.40 ± 0.05	44.86 ± 0.32	29.91 ± 0.51	112.1 ± 0.61	37.38 ± 0.44	24.90 ± 0.20	281.6 ± 1.2
7c	50.69 ± 0.61	34.61 ± 0.27	17.30 ± 0.04	92.72 ± 0.47	28.43 ± 0.37	40.18 ± 0.51	264.0 ± 1.7
8a	62.98 ± 0.45	53.29 ± 0.42	25.43 ± 0.64	17.25 ± 0.04	33.30 ± 0.41	27.25 ± 0.31	272.3 ± 1.1
8b	36.37 ± 0.34	12.12 ± 0.33	13.33 ± 0.06	12.12 ± 0.03	48.49 ± 0.60	16.62 ± 0.05	183.1 ± 0.6
8c	29.62 ± 0.21	24.69 ± 0.50	59.25 ± 0.76	29.62 ± 0.12	37.03 ± 0.42	21.60 ± 0.03	201.8 ± 0.9
9a	27.03 ± 0.13	49.23 ± 0.52	35.15 ± 0.04	81.24 ± 0.64	55.39 ± 0.42	52.31 ± 0.71	303.4 ± 1.0
9b	42.22 ± 0.37	37.25 ± 0.24	24.83 ± 0.11	99.35 ± 0.83	31.04 ± 0.53	27.94 ± 0.50	262.7 ± 0.5
9c	287.2 ± 2.10	12.04 ± 0.10	66.26 ± 0.63	93.97 ± 0.53	33.13 ± 0.04	21.08 ± 0.07	515.7 ± 1.7
10a	46.29 ± 0.70	37.03 ± 0.70	27.77 ± 0.32	115.7 ± 0.74	57.89 ± 0.08	57.87 ± 0.53	342.6 ± 1.4
10b	33.80 ± 0.40	31.38 ± 0.30	26.55 ± 0.06	96.75 ± 0.44	36.21 ± 0.33	51.30 ± 0.42	275.8 ± 1.3
10c	27.39 ± 0.53	48.99 ± 0.44	34.29 ± 0.16	17.54 ± 0.34	42.87 ± 0.51	51.31 ± 0.87	180.0 ± 0.7
